# Adverse events in single-arm clinical trials with non-fatal time-to-event efficacy endpoint: from clinical questions to methods for statistical analysis

**DOI:** 10.1186/s12874-023-02123-z

**Published:** 2024-01-03

**Authors:** Elena Tassistro, Davide Paolo Bernasconi, Maria Grazia Valsecchi, Laura Antolini

**Affiliations:** 1 Bicocca Center of Bioinformatics, Biostatistics and Bioimaging (B4 centre), School of Medicine and Surgery, University of Milano-Bicocca, Monza, Italy; 2 Biostatistics and Clinical Epidemiology, Fondazione IRCCS San Gerardo dei Tintori, Monza, Italy

**Keywords:** Survival analysis, Adverse events, Competing risks, Dependent censoring, IPCW

## Abstract

**Background:**

In any single-arm trial on novel treatments, assessment of toxicity plays an important role as occurrence of adverse events (AEs) is relevant for application in clinical practice. In the presence of a non-fatal time-to-event(s) efficacy endpoint, the analysis should be broadened to consider AEs occurrence in time.

The AEs analysis could be tackled with two approaches, depending on the clinical question of interest. Approach 1 focuses on the occurrence of AE as first event. Treatment ability to protect from the efficacy endpoint event(s) has an impact on the chance of observing AEs due to competing risks action. Approach 2 considers how treatment affects the occurrence of AEs in the potential framework where the efficacy endpoint event(s) could not occur.

**Methods:**

In the first part of the work we review the strategy of analysis for these two approaches. We identify theoretical quantities and estimators consistent with the following features: (a) estimators should address for the presence of right censoring; (b) theoretical quantities and estimators should be functions of time.

In the second part of the work we propose the use of alternative methods (regression models, stratified Kaplan-Meier curves, inverse probability of censoring weighting) to relax the assumption of independence between the potential times to AE and to event(s) in the efficacy endpoint for addressing Approach 2.

**Results:**

We show through simulations that the proposed methods overcome the bias due to the dependence between the two potential times and related to the use of standard estimators.

**Conclusions:**

We demonstrated through simulations that one can handle patients selection in the risk sets due to the competing event, and thus obtain conditional independence between the two potential times, adjusting for all the observed covariates that induce dependence.

## Introduction and rationale

The evaluation of outcome following a novel therapeutic regimen commonly considers a primary, possibly composite time to event(s) endpoint, related to disease control and survival. However, the assessment of toxicity plays an important role as the occurrence of severe adverse events (AEs) (or reactions) that may be disabling (although not deadly) is required for a careful treatment application in clinical practice. As an example, frontline intensive chemotherapy in children newly diagnosed with acute lymphoblastic leukemia (ALL) has remarkably increased in the last decades the ability to avoid relapse of the disease. Nowadays, more attention is given, when innovating the therapeutic approach, or applying it in clinical practice, to avoid or prevent undesirable disabling conditions (such as severe osteonecrosis) [1]. Thus, the analysis of outcome is broadened to evaluate and describe the occurrence of AEs such as osteonecrosis in addition to the primary endpoint of efficacy, such as relapse. This means that treatment failure considers the first event occurring between AE and relapse, thus placing the analysis in the context of competing risks. Yet, the occurrence of a relapse as first event does not exclude the possibility of observing a subsequent AE, but the relationship to the treatment under analysis is weakened because the patient undergoes a different treatment for relapse [2]. As a consequence, the occurrence of relapse is considered as a competing risk, thus as a sort of right censoring. Another possibility when in the presence of a non-fatal time-to-event efficacy endpoint (such as relapse) is the addition of a lag time where if the AE occurs within the lag time it can be still considered related to the initial treatment. For simplicity, we did not consider that lag time. Descriptive methods commonly used to analyse AEs data are the crude proportion of AEs, obtained by dividing the observed number of AEs by the total number of subjects, and the classical epidemiological AEs rate, obtained by dividing the observed number of AEs by the total time spent free from treatment failure. These measures are commonly reported in clinical papers among the initial descriptive results [3–6].

In principle, the analysis of AEs could be tackled from two different points of view. Approach 1 requires a competing risk framework for analysis: the clinical question relates to the observed occurrence of AE as first event, in the presence of the event “relapse”. In this case, AE and relapse are competing events, and treatment ability to protect from relapse has an impact on the chance of observing AEs due to the competing risks action [5].

Approach 2 requires a potential (or direct) framework for analysis: the clinical question relates to the treatment causing AE occurrence as if relapse could not occur. In this case, one should consider the occurrence of AEs as if relapse would not exclude the possibility of observing AEs related to the treatment under analysis, thus in the absence of competing risks [7]. These two approaches have very different implications when the description of AEs (and relapse) occurrence is used to comparatively describe two different treatment approaches (novel vs standard, for example). Indeed, in approach 1, the more one of the treatments protects from relapse, the greater is the chance of observing an AE as first event [5]. On the other hand, with approach 2, the effect of treatment on relapse is ruled out, in principle.

Regardless of the approach of interest, estimators and theoretical quantities used in clinical papers to describe AEs data should have the following features:

- estimators should address for the presence of right censoring;
- theoretical quantities and estimators should be functions of time.

This work has two aims: the first is to critically review the standard theoretical quantities and estimators with reference to their appropriateness for dealing with approaches 1 or 2 and to the desired features (a) and (b). The second aim is to define a strategy to relax the assumption of independence between the potential times to the competing events, such as that to AE and that to relapse, of the commonly used estimators when potential approach 2 is of interest.

The paper is organized as follows: in “Notation, setting and simulated example data” section we define notation and setting and we introduce a simulated dataset that will be used to present the standard methods. In “Standard methods” section we review the standard methods: the crude proportion of AEs and the epidemiological AEs rate, which fail in at least one of the two desirable features; the crude incidence and cause-specific estimators which are instead consistent with the two features (a) and (b). In “The potential approach estimation of the cumulative incidence of AE” section we clarify the impact of the crucial assumption of independence between potential times to competing events (AEs and relapse) of the standard estimators used in the potential approach. We propose the use of regression models, stratified Kaplan-Meier curves and inverse probability of censoring weighting to relax the assumption of independence by achieving conditional independence given covariates. In “Motivating example: osteonecrosis in childhood acute lymphoblastic leukemia” section we present results of the standard and regression-based methods on the motivating example on osteonecrosis in childhood ALL. “Simulation protocol” section describes an extensive simulation protocol developed to show the performance of the proposed methods and the impact of not accounting for an unmeasured covariate and “Simulation results” section presents the results of the simulations. The paper ends with discussion in “Discussion and conclusion” section.

## Notation, setting and simulated example data

The occurrence of AEs in time defines a survival time from origin $T_{AE}$. Similarly, the occurrence of a relapse in time defines the survival time $T_{RL}$. These survival times are called “potential” since only the minimum is observable as first event. The observable failure time is $T=min(T_{AE},T_{RL})$ and the observable cause of failure is *E* (equal to 1 if AE, equal to 2 if relapse). In the presence of a right censoring time *C*, the observed time is *min*(*T*, *C*) and $\Delta =I(T \le C)$ is the failure indicator. Finally, $(t_i, \delta _i, \delta _i \cdot e_i), i=1,...,N$, is used to denote the observed failure time, the failure indicator and the cause of failure, on a sized *N* sample.

In order to make the standard methods commonly used to analyse AEs data clearer, we simulated an example data of $N=300$ subjects using the inversion method of Bender et al. [8]. The potential times $T_{AE}$ and $T_{RL}$ were simulated from exponential distributions with parameters depending on two independent binary covariates $X_1$ and $X_2$, with $P(X_1 = 1) = 0.3$ and $P(X_2 = 1) = 0.4$. The combination of $X_1$ and $X_2$ identifies a different hazard profile in patients experiencing an AE or a relapse:

- if $X_1 = 0$ and $X_2 = 0, T_{AE} \sim Exp(1)$ and $T_{RL} \sim Exp(2)$
- if $X_1 = 0$ and $X_2 = 1, T_{AE} \sim Exp(3)$ and $T_{RL} \sim Exp(6)$
- if $X_1 = 1$ and $X_2 = 0, T_{AE} \sim Exp(3)$ and $T_{RL} \sim Exp(5)$
- if $X_1 = 1$ and $X_2 = 1, T_{AE} \sim Exp(9)$ and $T_{RL} \sim Exp(15)$

**Fig. 1:**
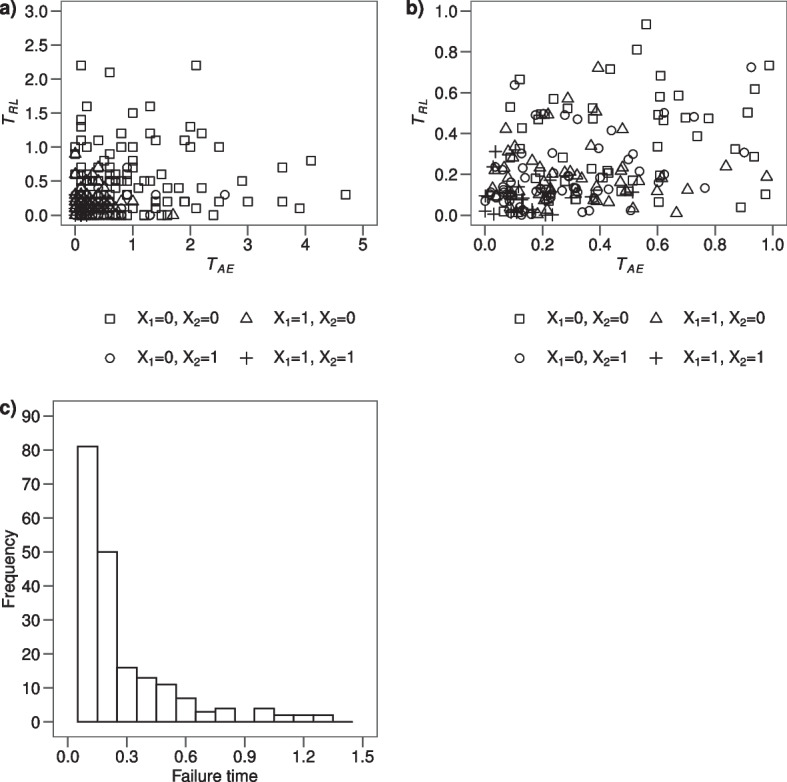
**a** Scatterplot of the potential times $T_{AE}$ and $T_{RL}$ according to the four groups identified by the binary covariates $X_1$ and $X_2$; **b** Zoom of the scatterplot in panel a) selecting potential times $T_{AE}$ and $T_{RL}$ lower than 1; **c** histogram of the distribution of the failure times calculated as the minimum value between $T_{AE}$ and $T_{RL}$, in the simulated data of $N=300$ subjects

One may note that, fixed $X_1$ (e.g. $X_1 = 0$ or $X_1 = 1$), if $X_2$ changes from 0 to 1, both the hazard of AE and the hazard of relapse triple. For sake of simplicity, we do not consider the presence of censoring, but we will explain the influence of right censoring on each standard method. The distribution of $T_{AE}$ and $T_{RL}$ is presented in Fig. 1 panel a) and b).

At first glance, the dependence between times $T_{AE}$ and $T_{RL}$ is not evident. As expected, the times of patients with both covariates equal to 0 are systematically greater than the remaining ones. Indeed, median values for $T_{AE}$ and $T_{RL}$ are: 0.7 and 0.4 ($X_1 = 0, X_2 = 0$), 0.2 and 0.1 ($X_1 = 0, X_2 = 1$), 0.2 and 0.2 ($X_1 = 1, X_2 = 0$), 0.1 and 0.0 (both covariates equal to 1) with a Spearman correlation equal to 0.95. However the overall Spearman correlation between times $T_{AE}$ and $T_{RL}$ is equal to 0.25, a moderate value due to the absence of correlation within each of the four groups of patients conditional on the covariate value identifying the groups. The distribution of the failure times *T*, calculated as the minimum value between $T_{AE}$ and $T_{RL}$, is displayed in Fig. 1 panel c). The distinct failure times $t_j$, the number of patients at risk $n_j$ at each time point and the number of subjects experiencing an AE or a relapse as first event ($d_{jAE}$ and $d_{jRL}$ respectively) are displayed in Table 1, where 80 subjects developed an AE and 220 relapsed.

**Table 1:** Simulated example data of $N=300$ subjects and estimators

*j*	$t_j$	$n_j$	$d_{jAE}$	$d_{jRL}$	$\hat{S}(t_j)$	$\hat{h}_{AE}(t_j)$	$\hat{h}_{AE}(t_j)\hat{S}(t_j-)$	$\hat{CI}_{AE}(t_j)$	$\hat{AN}_{AE}(t_j)$	$\hat{KM}_{AE}(t_j)$
1	0.0	300	29	75	0.653	0.097	0.097	0.097	0.097	0.097
2	0.1	196	21	60	0.383	0.107	0.070	0.167	0.204	0.194
3	0.2	115	11	39	0.217	0.096	0.037	0.204	0.300	0.271
4	0.3	65	4	12	0.163	0.062	0.013	0.217	0.362	0.316
5	0.4	49	6	7	0.120	0.122	0.020	0.237	0.484	0.400
6	0.5	36	1	10	0.083	0.028	0.003	0.240	0.512	0.416
7	0.6	25	3	4	0.060	0.120	0.010	0.250	0.632	0.486
8	0.7	18	0	3	0.050	0.000	0.000	0.250	0.632	0.486
9	0.8	15	2	2	0.037	0.133	0.007	0.257	0.765	0.555
10	1.0	11	1	3	0.023	0.091	0.003	0.260	0.856	0.595
11	1.1	7	0	2	0.017	0.000	0.000	0.260	0.856	0.595
12	1.2	5	0	2	0.010	0.000	0.000	0.260	0.856	0.595
13	1.3	3	1	1	0.003	0.333	0.003	0.263	1.189	0.730
14	2.1	1	1	0	0.000	1.000	0.003	0.266	2.189	1.000

$t_j$ are the distinct failure times measured in years; $n_j$ is the number of patients at risk at time $t_j$; $d_{jAE}$ and $d_{jRL}$ are the number of patients developing AE or relapse at time $t_j$, respectively; $\hat{S}(t_j)$ is the survival function at time $t_j$, estimated according to KM estimator on failures due to AE or relapse; $\hat{h}_{AE}(t_j)$ corresponds to the instantaneous rate of AE and it is the non-parametric ML estimator of the $CSH_{AE}(t)$; $\hat{h}_{AE}(t_j)\hat{S}(t_j-)$ is the product of instantaneous rate of AE with the KM estimator of the proportion of patients free from treatment failure up to time $t_j-$; $\hat{CI}_{AE}(t_j)$ is the AJ estimator of $CI_{AE}(t)$ of AE; $\hat{AN}_{AE}(t_j)$ and $\hat{KM}_{AE}(t_j)$ are the AN and KM estimates of the $CSH_{AE}(t)$

## Standard methods

### The crude proportion of AE

The empirical crude proportion (CP) is defined as

$$\begin{aligned} CP = \sum \limits _{i=1}^{N} \frac{I(\delta _i\cdot e_i=1)}{N} \end{aligned}$$

where $I(\cdot )$ is the indicator function. CP is the count of patients who fail due to AEs during the entire follow-up over a total of *N* patients, regardless of the individual follow-up length.

In our example data, this quantity is $CP=\frac{80}{300}=0.27$. CP is consistent with the first approach of analysis, since it can be thought as a naïve estimate of the probability of observing AEs over the entire follow-up. Of note, AEs are counted in the numerator of CP only if observed as first events, and relapse acts as competing risk. CP is not a function of time in the sense that is not calculated at different time points and it does not address properly for the presence of right censoring that affects the count in the numerator, but *N* is fixed. Thus, CP fails with respect to both features (a) and (b).

### The crude incidence of AE

**Fig. 2:**
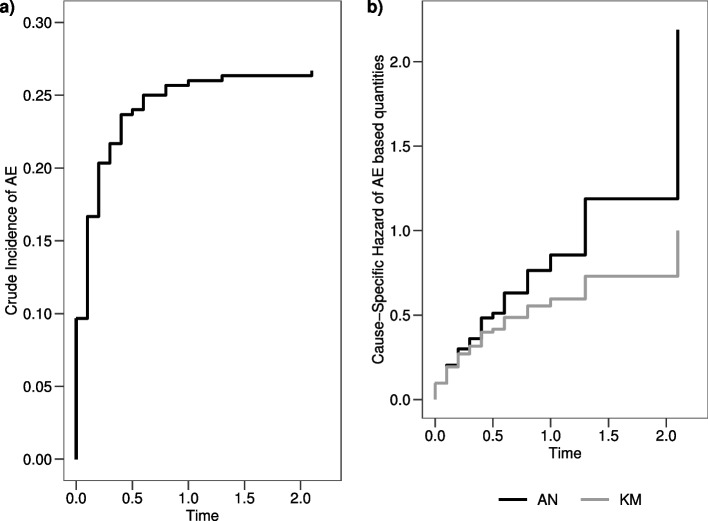
**a** $CI_{AE}(t)$ estimated through the AJ formula; **b** $AN_{AE}(t)$ and $KM_{AE}(t)$ step curves of the cumulative $CSH_{AE}(t)$ and of the cumulative incidence of AE, respectively

The theoretical CP can be generalized in time by the crude cumulative incidence (CI) probability (that in the remaining of the work will be called crude incidence) $CI_{AE}(t)=P(T \le t; E=1)$, which corresponds to the absolute risk of treatment failure due to AEs up to time *t*. The non-parametric maximum likelihood (ML) estimator of $CI_{AE}(t)$ is given by the Aalen-Johansen (AJ) formula

1 $$\begin{aligned} \hat{CI}_{AE}(t)= \sum \limits _{t_j \le t} \hat{S}(t_j-) \cdot \hat{h}_{AE}(t_j) \end{aligned}$$

where $\hat{S}(t_j-)=\hat{P}(T>t_j-)$ is the KM non-parametric ML estimator of the proportion of patients free from treatment failure, either caused by AE or relapse, up to time $t_j-$ and

2 $$\begin{aligned} \hat{h}_{AE}(t) =\frac{\sum _{i=1}^{N} I(t_i=t_j;\delta _{ij} \cdot e_{ij}=1)}{n_j} \end{aligned}$$

is the instantaneous rate of AEs, which corresponds to the proportion of patients experiencing AEs at time *t* over the total number of patients at risk, i.e. free from AEs and relapse (and censoring), at that time. Of note, the denominator corresponds to the person-time spent at risk in the time window $$[t_j, t_j+1)$$. In Table 1, at each time $$t_j$$, the quantities needed to estimate $$\hat{CI}_{AE}(t)$$ are displayed and in Fig. 2 panel a) the graph is shown.

The AJ estimator of $CI_{AE}(t)$ is consistent with the first approach of analysis, since it can be thought as an estimate of the probability of treatment failure due to AEs over the course of time, where, since AEs are counted only if observed as first events, relapse acts as a competing event. One may note in (1) the indirect protection of relapse, that lowers down $\hat{S}(t_j-)$ when relapse occurs. The $CI_{AE}(t)$ has both features (a) and (b): it addresses for the presence of right censoring, due to the non-parametric ML estimator property, and it is a function of time. In this setting, the possible impact of covariates on the $CI_{AE}(t)$ can be assessed by the Fine and Gray model or the regression model based on pseudo-values.

### The epidemiological AE rate

The epidemiological AE rate is defined as

$$\begin{aligned} Rate= \frac{\sum _{i=1}^{N}I(\delta _i\cdot e_i=1)}{\sum _{i=1}^{N} t_i} \end{aligned}$$

and it originates from the count of patients observed to fail due to AEs during the entire follow-up divided by the total time spent free from treatment failure, i.e. spent free both from AEs and relapse. The AE rate represents the number of observed AEs per 1 unit of person-time spent at risk.

In our example, the AE rate is $Rate=\frac{80}{56.4\cdot12}=0.12$ with the total time at the denominator calculated in months from Table 1. The AE rate can be thought as an estimate of the probability of observing AEs in the next time unit for a patient that is now free from AEs and relapse (and censoring), assuming this probability constant in time. If this probability cannot be reasonably assumed constant, the AE rate can be interpreted as an “average” rate over the follow-up. The AE rate is consistent with the second approach of analysis, where the focus is on treatment action in the development of AEs in patients relapse free in time. Indeed, the occurrence of relapse (or of right censoring) implies a contribution to the denominator of the time to relapse (or to right censoring) and a null contribution to the numerator. The AE rate addresses feature (a) but not (b), due to the assumption of constancy in time. The AE rate can be proved to be the parametric ML estimator of the probability of observing AEs in the next time unit for a patient free from treatment failure (either caused by relapse or AE) assuming this probability constant in time.

### The cause-specific hazard of AE and related quantities

The theoretical AE rate can be easily generalized in time by relaxing the assumption of constancy in time by

3 $$\begin{aligned} CSH_{AE}(t)= \lim _{\Delta t\rightarrow 0^+} \frac{P(t<T \le t+\Delta t; E=1| T>t)}{\Delta t} \end{aligned}$$

This quantity corresponds to the cause-specific hazard (CSH) of AEs and the estimator of the instantaneous hazard is $\hat{h}_{AE}(t)$ in (2). Based on cause-specific hazard, two important cumulative step function estimators can be derived, as described below.

#### The Aalen-Nelson estimator of the cumulative hazard of AE

We may consider the cumulative sum of $CSH_{AE}(t)$ to obtain an estimator of the cumulative hazard $\int _{0}^{t} CSH_{AE}(u)du$ by the Aalen-Nelson (AN) formula

4 $$\begin{aligned} \hat{AN}_{AE}(t)= \sum \limits _{t_j \le t} 1 \cdot \hat{h}_{AE}(t_j) \end{aligned}$$

which is based on the non-parametric ML estimator $\hat{h}_{AE}(t)$ in (2). The $AN_{AE}(t)$ estimator is consistent with the second approach of analysis, as there is no indirect protection from the competing event (relapse). One may observe by comparing (1) and (4) that $\hat{S}(t_j-)$, which is lowered down in (1) when a relapse occurs, is replaced in (4) by the fixed value 1, as if relapses were removed.

Estimator (4) addresses for the presence of right censoring (feature (a)) since $\hat{h}_{AE}(t_j)$ does so, and it is a function of time (feature (b)). The values of the estimates are reported in Table 1 and the corresponding curve is displayed in Fig. 2 panel b).

The $AN_{AE}(t)$ curve can be interpreted as a naïve estimator of the expected number of AEs a patient may experience in the time interval up to *t* when he/she can develop recurrent AEs in time as if relapse was removed.

#### The Kaplan-Meier estimator of the cumulative incidence of AE

One may consider the cumulative product of $(1-\hat{h}_{AE}(t))$ terms, with the complement to 1 to obtain an estimator of the cumulative incidence function that corresponds to $1-\exp \left( \int _{0}^{t} -CSH_{AE}(u)du \right)$, by the KM formula

5 $$\begin{aligned} \hat{KM}_{AE}(t)= 1 - \prod _{t_j \le t} (1- \hat{h}_{AE}(t_j) ) \end{aligned}$$

which is based on the non-parametric ML estimator $\hat{h}_{AE}(t)$ in (2).

Estimator (5) addresses for the presence of right censoring (feature (a)) since it is addressed in $\hat{h}_{AE}(t_j)$, and it is a function of time (feature (b)). In Table 1 the values of the estimates obtained with this method are reported and the corresponding curve is displayed in Fig. 2 panel b).

The $\hat{KM}_{AE}(t)$ curve can be interpreted as a naïve estimator of the cumulative incidence of treatment failure only due to AEs, as if relapse was removed. This $\hat{KM}_{AE}(t)$ corresponds to a KM estimator where relapse is just a censored observation. Also, $1-\hat{KM}_{AE}(t)$ is often used to naïvely estimate the so called “*AE free survival curve*” that is the cumulative probability of not having AEs in time (censoring time to relapse).

## The potential approach estimation of the cumulative incidence of AE

### Issues on the meaning of AN and KM estimators

At first glance, the $AN_{AE}(t)$ and $KM_{AE}(t)$ curves could be interpreted only in terms of treatment action in determining AE occurrence, regardless of the impact of relapse. This interpretation comes natural since $h_{AE}(t)$ is related only to the velocity of development of AEs in time. It is not so for the crude incidence $CI_{AE}(t)$, where the presence of $S(t_j-)$ in (1) makes evident that the occurrence of relapse has a direct influence on the estimate.

One may note, however, that the occurrence of relapse may exclude “not at random” patients from the risk set on which the instantaneous rate of AEs is calculated in (2). This indirect patients selection due to relapse may influence the interpretation of $CSH_{AE}(t)$ and of the chosen step function. Due to this, the $CSH_{AE}(t)$ in (3) may not capture entirely how treatment influences the occurrence of AE at time *t* in patients who, at that time, would be free from AE, which is theoretically represented by the potential hazard (pH) of AE

6 $$\begin{aligned} pH_{AE}(t)=\lim _{\Delta t\rightarrow 0^+} \frac{P(t<T_{AE} \le t+\Delta t|T_{AE}>t)}{\Delta t} \end{aligned}$$

with corresponding potential cumulative incidence

$$\begin{aligned} pI_{AE}(t)=P(T_{AE}\le t) \end{aligned}$$

that represents treatment action on AE, regardless of the impact of relapse.

Only if there is independence between $T_{AE}$ and $T_{RL}$, the sub-sample of patients with $T>t$ in (3) is a random sample of patients with $T_{AE}>t$ in (6) and thus the two expressions (3) and (6) coincide. As a consequence, under the assumption of independence, $\hat{KM}_{AE}(t)$ in (5) is the suitable estimator of $pI_{AE}(t)$. A similar argument follows for $\hat{AN}_{AE}(t)$ as estimator of $\int _{0}^{t} pH_{AE}(u)du$ This assumption, however, is often not reasonable and cannot be tested.

To enlighten the problems related to the interpretation of $\hat{AN}_{AE}(t)$ and $\hat{KM}_{AE}(t)$ in the absence of independence, we simulated different datasets according to the setting specified in “Notation, setting and simulated example data” section, with increasing sample size from $N=100$ to $N=1000$, and we compared these quantities with the potential cumulative hazard function $pH_{AE}(t)$ and incidence function $CFI_{AE}(t)$. The potential cumulative hazard function can be calculated as $-log(pS_{AE}(t))$, where the potential survival function $pS_{AE}(t)=1-pI_{AE}(t)$ defined as

7 $$\begin{aligned} pS_{AE}(t){} & {} = P(X_1=0) P(X_2=0) exp(-\lambda _{00AE}\cdot t) + \nonumber \\{} & {} + P(X_1=0) P(X_2=1) exp(-\lambda _{01AE}\cdot t) + \nonumber \\{} & {} + P(X_1=1) P(X_2=0) exp(-\lambda _{10AE}\cdot t) + \nonumber \\{} & {} + P(X_1=1) P(X_2=1) exp(-\lambda _{11AE}\cdot t) \end{aligned}$$

corresponds to an average of exponential distributions weighted by the proportion of patients with covariates values $X_1=k, k=0,1$ and $X_2=l, l=0,1$.

**Fig. 3:**
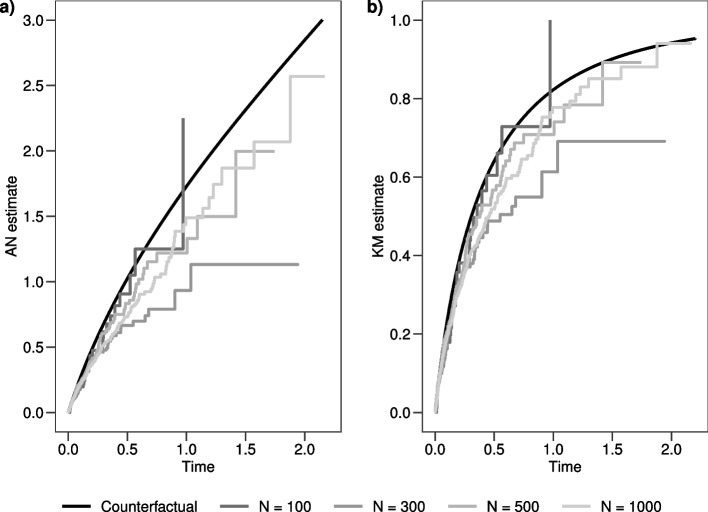
**a** $\hat{AN}_{AE}(t)$ calculated for different sample sizes; **b** $\hat{KM}_{AE}(t)$ calculated for different sample sizes

In Fig. 3 panels a) and b) the $\hat{AN}_{AE}(t)$ and $\hat{KM}_{AE}(t)$ curves calculated in the simulated datasets are displayed together with the potential quantities. One can notice that, independently from the sample size of the dataset and from the low overall Spearman correlation between times $T_{AE}$ and $T_{RL}$ (0.17, 0.33, 0.20, 0.26 for sample sizes $N=100, N=300, N=500, N=1000$ respectively), the $\hat{AN}_{AE}(t)$ and $\hat{KM}_{AE}(t)$ curves do not fit the potential quantities.

To relax the assumption of independence between the two potential times $T_{AE}$ and $T_{RL}$ one possibility is to estimate the hazard of AE in strata defined by covariates (that influence the AE and relapse time distributions) assuming that only within each stratum there is independence between $T_{AE}$ and the indirect selection due to $T_{RL}$. This approach can be carried out by averaging stratum estimates obtained either non parametrically or by the use of the Cox regression model on $CSH_{AE}(t)$ leading to a weighted average survival probability. An alternative method is addressing the presence of selection due to relapse through inverse probability of censoring weighting (IPCW) [9–11]. This method aims at creating a pseudo-population that is similar to the one observable in the absence of relapse by adding a weight to patients who do not develop relapse. On this pseudo-population a survival probability is then calculated and the incidence is derived as its complement to 1.

### Weighted average survival probability

The survival probabilities for each combination of the observed covariates values $pS^{kl}_{AE}(t)=P(T_{AE}>t|X_1=k,X_2=l), k=0,1$ and $l=0,1$, can be estimated through the KM estimator within each stratum or by a Cox proportional hazards (PH) model including these covariates among regressors:

$$\begin{aligned} CSH_{AE}(t)=CSH_{0,AE}(t) \exp (\beta _1 X_1+\beta _2 X_2) \end{aligned}$$

where $CSH_{0,AE}(t)$ is the baseline hazard.

The overall average survival is determined by weighting the survival probabilities in each level of the covariates by the proportion of subjects having that covariate levels. If both covariates $X_1$ and $X_2$ are observed, the weighted average survival is calculated as

8 $$\begin{aligned} \hat{pS}_{AE}(t){} & {} = \frac{\sum _{i=1}^{n} I(X_{1i}=0, X_{2i}=0)}{n} \hat{pS}^{00}_{AE}(t) + \nonumber \\{} & {} + \frac{\sum _{i=1}^{n} I(X_{1i}=0, X_{2i}=1)}{n} \hat{pS}^{01}_{AE}(t) + \nonumber \\{} & {} + \frac{\sum _{i=1}^{n} I(X_{1i}=1, X_{2i}=0)}{n} \hat{pS}^{10}_{AE}(t) + \nonumber \\{} & {} + \frac{\sum _{i=1}^{n} I(X_{1i}=1, X_{2i}=1)}{n} \hat{pS}^{11}_{AE}(t) \end{aligned}$$

where $\hat{pS}^{kl}_{AE}(t)$ indicates the survival probability at time *t* obtained through the KM estimator or predicted by the Cox model for a patient having $X_1=k, k=0,1$, and $X_2=l, l=0,1$. This is an estimator of the AE free survival probability curve and the complement to 1 of this estimator represents the incidence probability.

### IPCW estimator of the survival probability

The unitary contribution of a subject *i* in the count of subjects at risk of experiencing an AE at time *t* is replaced in (2) and (5) by the weight $w_{i}(t) = \frac{1}{\hat{pS}_{RL}^{X_{1i},X_{2i}}(t)}$ where $\hat{pS}_{RL}^{X_{1i},X_{2i}}(t)$ is the estimate of the potential conditional probability $pS_{RL}^{X_{1},X_{2}}(t)=P(T_{RL}> t|X_{1},X_{2})$ of being relapse free until time *t*. The lower is the probability of being relapse free, the greater are the weights, given the inverse proportionality. The estimate of $pS_{RL}^{X_{1},X_{2}}(t)$ can be based on the KM estimator within each combination of the observed covariates or on the fit of a Cox PH model for relapse $CSH_{RL}(t)=CSH_{0,RL}(t) \exp (\beta _1 X_1+\beta _2 X_2)$ in which prognostic factors for AE and relapse are entered as covariates. Alternative survival regression models can also be considered as for example the Aalen additive model which does not rely on the PH assumption [12]. Once the weights are calculated, one can estimate the survival probability for time to AE in the absence of relapse, i.e. the AE free survival curve, using the KM estimator [13] and then derive the incidence probability as its complement to 1.

## Motivating example: osteonecrosis in childhood acute lymphoblastic leukemia

We show here the application of methods revised in “Standard methods” section and of those proposed in “The potential approach estimation of the cumulative incidence of AE” section to data on children with ALL enrolled in two subsequent multicenter clinical trials conducted in Italy with the Italian Association of Pediatrical Hematology and Oncology (AIEOP) [1]. The aim is to assess how front line chemotherapy treatment is related or affects children with a relatively rare, yet disabling, complication such as severe osteonecrosis (ON). The primary evaluation of outcome usually considers a composite time-to-event endpoint, i.e. time to failure (where failure is defined as resistance, relapse, death or second malignant neoplasm, whichever occurs first). The analysis with focus on ON broadens the evaluation of treatment efficacy to relevant toxic events, as if this relevance would justify considering them as failures, in other words as competing events. In particular, here ON is an AE of the frontline treatment protocol administered to children with ALL and acts as competing risk for death (not due to relapse) or first relapse (which is followed by another type of treatment).

We analysed data on 3691 children aged 1-17 years at diagnosis of ALL and, of those, 99 experienced an ON during or after the end of the front line treatment while 725 children experienced a competing event of the primary endpoint (596 developed a relapse, 96 died, 23 experienced a second malignant tumour and 10 were resistant); all others were right censored at last follow-up. In the following, we will identify the competing event as relapse or death.

**Fig. 4:**
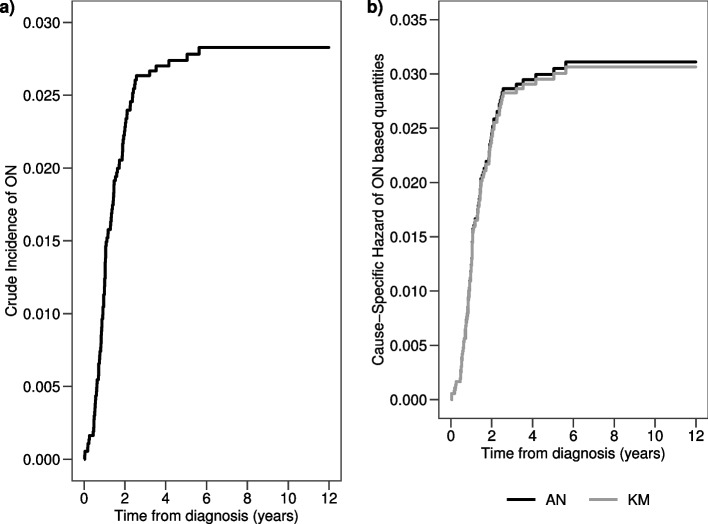
**a**) $CI_{ON}(t)$ estimated through the AJ formula; **b**) $AN_{ON}(t)$ and $KM_{ON}(t)$ step curves of the cumulative $CSH_{ON}(t)$ and of the cumulative incidence of ON, respectively

The crude proportion of ON is $CP=\frac{99}{3691}=0.027$, meaning that $2.7\%$ of the study population developed an AE before relapse or death. In Fig. 4 panel a) the crude incidence of ON calculated through the AJ estimator is displayed. The CI probability of failure due to an ON is lower than $3.0\%$ after 6 years from diagnosis of ALL.

The epidemiological AE rate, calculated as the number of subjects experiencing an ON over the total time at risk of developing an ON (i.e. time at risk is time to ON, relapse/death, censoring) is $Rate=\frac{99}{19940.96}=0.005$, meaning that 5 ONs per 1000 person-years occurred. The estimates of the $CSH_{ON}(t)$ obtained through the AN and KM estimators are displayed in Fig. 4 panel b). Multiplying by 100 the AN estimator at 6 years from diagnosis, the expected number of ONs in 100 hypothetical children is 3.11. The estimated cumulative incidence at 6 years is 0.0306 ($SE=0.003$) and it is close, as expected due to rarity, to the $CSH_{ON}(t)$ estimate (0.0311).

In this context two covariates are of relevance: age at diagnosis of ALL, since incidence of ON and of relapse tends to be higher with higher age, and risk group, which is the stratification of the children, based on genetic features and cytological/molecular early response to treatment, that defines the intensity of the administered treatment (the higher the risk, the higher the intensity). In order to correctly account fo the presence of a dependence between the potential time of ON and the potential time of relapse or death we derived the estimate of the potential incidence of ON from formula (8), including first only risk group as a covariate and then adding also age at diagnosis ($>10$ versus $\le 10$ years).

**Fig. 5:**
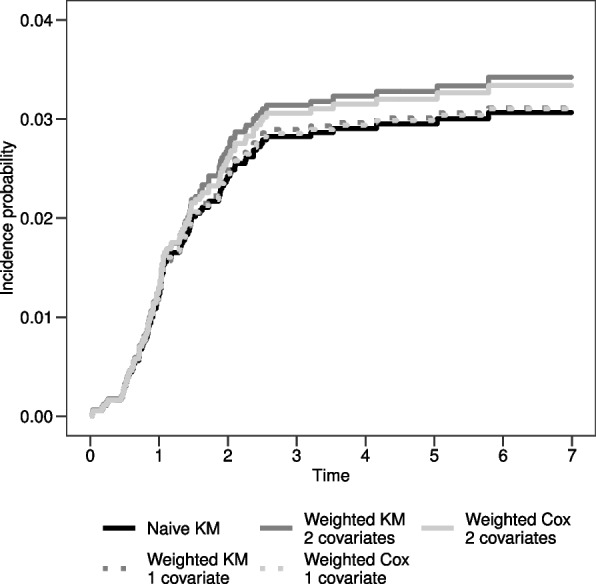
Incidence probability estimates obtained with the naïve KM estimator, the weighted KM estimator stratifying only for 1 covariate (risk group) or for 2 covariates (risk group and age at diagnosis) and the weighted Cox model including only risk group or both risk group and age at diagnosis as covariates

In Fig. 5 the estimates of the cumulative incidence probability obtained with different methods are displayed. One can see that the naïve KM estimator and the weighted average method considering only risk group as covariate give the same estimates of the incidence probability. Including also age at diagnosis as covariate, a similar but higher incidence probability is obtained. The distance between these two groups of curves suggests that the inclusion of the second covariate removes part of the dependence between time to ON and to relapse/death. However, overall the inclusion of the covariates does not change remarkably the incidence probability compared to the naïve estimator. This could be due to either to the low ability of the covariates to remove the dependence or to the fact that the two potential times do not have a strong dependence as the pathways to relapse or death are not so much related to the determinants of ON.

## Simulation protocol

### Data generation

We extended the simulation setting of “Notation, setting and simulated example data” section (1000 datasets with $N=300$ each) by considering 4 different scenarios [8, 14].

The 4 scenarios differ in the parameters of the exponential distribution from which the potential competing time $T_{RL}$ is generated (Table 2). In scenario 1 none of the covariates has an impact on the hazard of the potential time $T_{RL}$ and potential times $T_{AE}$ and $T_{RL}$ are independent. In the other three scenarios there is at least one covariate with an impact on the hazard of the potential time $T_{RL}$ and the hazard of the potential time $T_{AE}$ that generates dependence between these potential times. In particular, in scenario 2 only $X_1$ has an impact on both hazards whereas in scenarios 3 and 4 both covariates have an impact on both hazards (scenario 4 is the same of “Notation, setting and simulated example data” section).

**Table 2:** Parameters of the exponential distributions of the potential times $T_{AE}$ and $T_{RL}$

Scenario	AE				Relapse			
	$\lambda _{00AE}$	$\lambda _{01AE}$	$\lambda _{10AE}$	$\lambda _{11AE}$	$\lambda _{00RL}$	$\lambda _{01RL}$	$\lambda _{10RL}$	$\lambda _{11RL}$
1	1	3	3	9	2	2	2	2
2	1	3	3	9	2	2	5	5
3	1	3	3	9	2	6	5	5
4	1	3	3	9	2	6	5	15

$\lambda _{klAE}$ and $\lambda _{klRL}$ are the parameters of the exponential distributions of $T_{AE}$ and $T_{RL}$, respectively, when $X_1=k, k=0,1$, and $X_2=l, l=0,1$

We simulated also an additional scenario varying the imbalance of the covariates, with two cases, in scenario 4. In case A we set the parameters of the Bernoulli distributions from which the binary covariates $X_1$ and $X_2$ are generated in order to construct the “worst” situation that may happen when analysing real data, that is both covariates are very frequent in the population under study. To do so, we changed the Bernoulli parameters from the original $P(X_1 = 1) = 0.3$ and $P(X_2 = 1) = 0.4$ to $P(X_1 = 1) = P(X_2 = 1) = 0.5$. Then, in case B we set the Bernoulli parameters in order to obtain the “best” scenario, that is at least one covariate (here $X_2$) is rare in the study population. In this case, we kept fixed the prevalence of the first covariate $P(X_1 = 1) = 0.3$ and we changed that of the second one to $P(X_2 = 1) = 0.1$.

We added another simulation where the parameters of the exponential distributions of the hazard of relapse where changed in order to reduce the impact of the competing event in scenario 4. First we set the parameters in order to have, fixed $X_1=0$ (or $X_1=1$), when $X_2$ changes, an increase of 2 times in the hazard of relapse (case A) and then an increase of 1.5 times in the hazard of relapse (case B).

Simulations were carried out using the R software version 4.0.3 available at http://cran.r-project.org/.

### Estimated quantities

For each scenario, we calculated the potential incidence probability in an hypothetical world where relapse is absent deriving it from formula (7) at two fixed time-points ($t=0.2$ and $t=0.3$). In addition, we estimated the expected number of subjects at risk of developing an event (AE or relapse) at these time-points for an hypothetical sample of $N=300$ patients as

$$\begin{aligned}{} & {} N \cdot P(T_{RL}>t, T_{AE}>t) = \\ = N \cdot{} & {} [ P(X_1=0) P(X_2=0) exp(-\lambda _{00RL}\cdot t) exp(-\lambda _{00AE}\cdot t) + \\{} & {} + P(X_1=0) P(X_2=1) exp(-\lambda _{01RL}\cdot t) exp(-\lambda _{01AE}\cdot t) + \\{} & {} + P(X_1=1) P(X_2=0) exp(-\lambda _{10RL}\cdot t) exp(-\lambda _{10AE}\cdot t) + \\{} & {} + P(X_1=1) P(X_2=1) exp(-\lambda _{11RL}\cdot t) exp(-\lambda _{11AE}\cdot t) ] \end{aligned}$$

where the calculation of $P(T_{RL}>t,T_{AE}>t)$ in each stratum is obtained by the product of $P(T_{RL}>t)$ and $P(T_{AE}>t)$ due to the conditional independence, given covariates.

We compared the estimators that can be used to estimate the potential incidence probability for each dataset:

1. the naïve $KM_{AE}(t)$ presented in formula (5)
2. the complement to 1 of the weighted average survival probability, obtained through the KM estimator in strata defined according to the observed covariate $X_1$, thus considering covariate $X_2$ as unobserved
3. the complement to 1 of the weighted average survival probability, obtained through the KM estimator in strata defined according to the observed covariates $X_1$ and $X_2$
4. the complement to 1 of the weighted average survival probability, obtained through the Cox model with only the observed covariate $X_1$, thus considering covariate $X_2$ as unobserved
5. the complement to 1 of the weighted average survival probability, obtained through the Cox model with the observed covariates $X_1$ and $X_2$
6. the complement to 1 of the KM survival probability on the pseudo-population obtained by the IPCW estimator (Cox based) where weights are estimated according to the observed covariate $X_1$, thus considering covariate $X_2$ as unobserved
7. the complement to 1 of the KM survival probability on the pseudo-population obtained by the IPCW estimator (Cox based) where weights are estimated according to the observed covariates $X_1$ and $X_2$.

## Simulation results

**Fig. 6:**
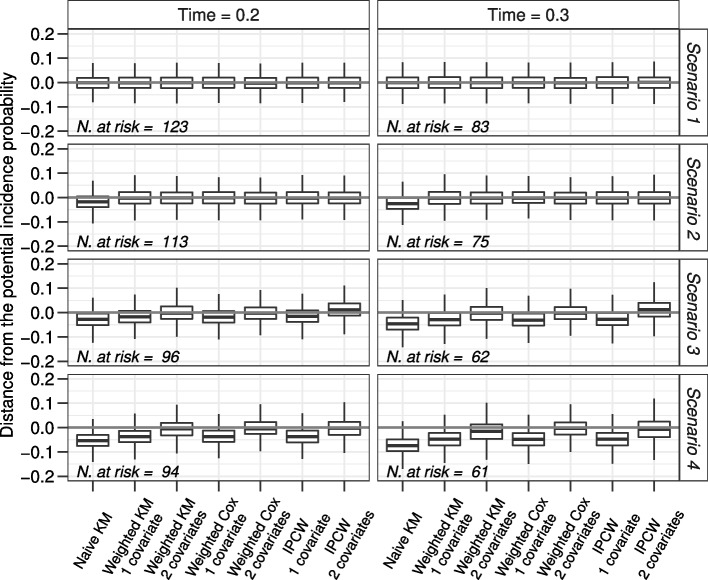
Simulation results in the four scenarios at times $t=0.2$ and $t=0.3$. The grey horizontal line is the reference null bias

Simulation results in the four scenarios are presented in Fig. 6. At each time-point the expected number of subjects at risk of developing an event is displayed and the distance between the estimate and the potential incidence probability (bias) is represented in a boxplot for each of the 7 estimators.

In scenario 1, where the hazard of relapse is independent from the covariates values, the results of all methods are similar: the median value of the bias of the incidence estimated through each method and the potential incidence is equal to 0. Of note, also the estimate of the incidence obtained with the naïve KM estimator censoring relapsed subjects is unbiased.

In scenario 2, where only $X_1$ has an impact on the hazard of relapse, the naïve KM estimator gives a biased incidence probability. With the other methods unbiased estimates are obtained. Of note, the methods in which only the $X_1$ covariate is considered perform slightly better with respect to those in which both covariates are included, due to the fact that $X_2$ does not have an impact on relapse.

**Fig. 7:**
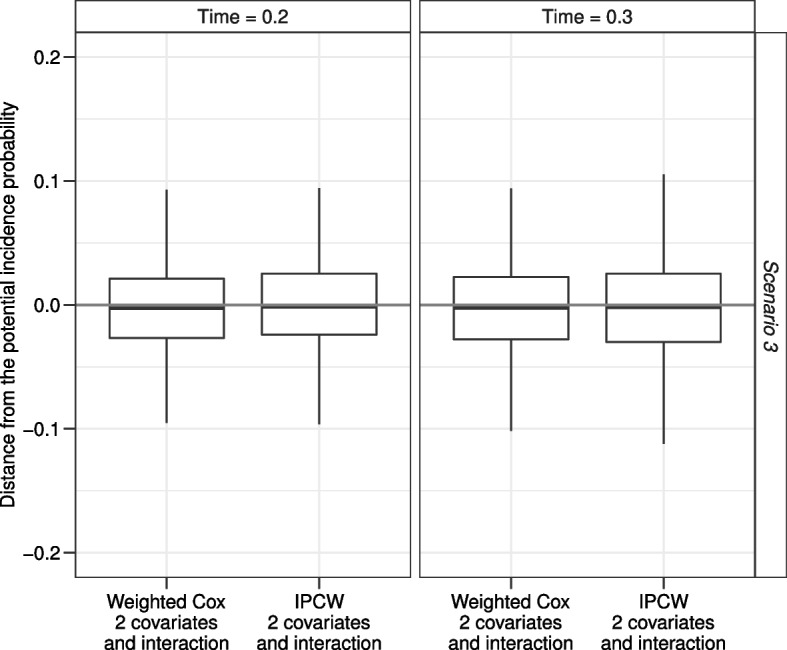
Simulation results for scenario 3 of the IPCW estimator accounting for the presence of an interaction between $X_1$ and $X_2$ in the estimate of the weights. The grey horizontal line is the reference null bias

In scenario 3 the hazard of relapse depends on $X_1$ and, when $X_1=0$, also on $X_2$. Methods in which only $X_1$ is included give biased estimates. Methods with $X_1$ and $X_2$ covariates give unbiased estimates with the exception of the IPCW estimator, where there is an overestimation of the incidence probability. This is due to the fact that in this scenario also an interaction between the two covariates is present: $X_2$ has an impact on the hazard of relapse only when $X_1=0$. However this interaction is not accounted for in the model to estimate weights. To corroborate this result, Fig. 7 shows the results of weighted Cox and IPCW approaches when $X_1, X_2$ and their interaction are considered. The reader may observe that the inclusion of the interaction overcomes the bias in the IPCW method, while it is not needed in the Cox model since, conditional on $X_1$ and $X_2$, this model requires an assumption of independence between $T_{AE}$ and $T_{RL}$ that is present even if there is an interaction between $X_1$ and $X_2$. In this regard, the Cox model is robust also in the presence of an interaction between the covariates on the hazard of relapse.

In the last scenario displayed in Fig. 6, when both $X_1$ and $X_2$ have an impact on the hazard of relapse, all methods including one covariate only give similar biased results. However, the distance between the estimated and the potential incidence probabilities is lower than that obtained with the naïve KM estimator. The estimates from the weighted KM or the Cox model and from the IPCW are unbiased when the methods account for the presence of all covariates that have an impact on the hazard of relapse. Of note, the estimates from the IPCW have a greater variability with respect to the others.

In the additional simulations on variations of scenario 4, all the proposed methods with the exception of the IPCW were compared.

**Fig. 8:**
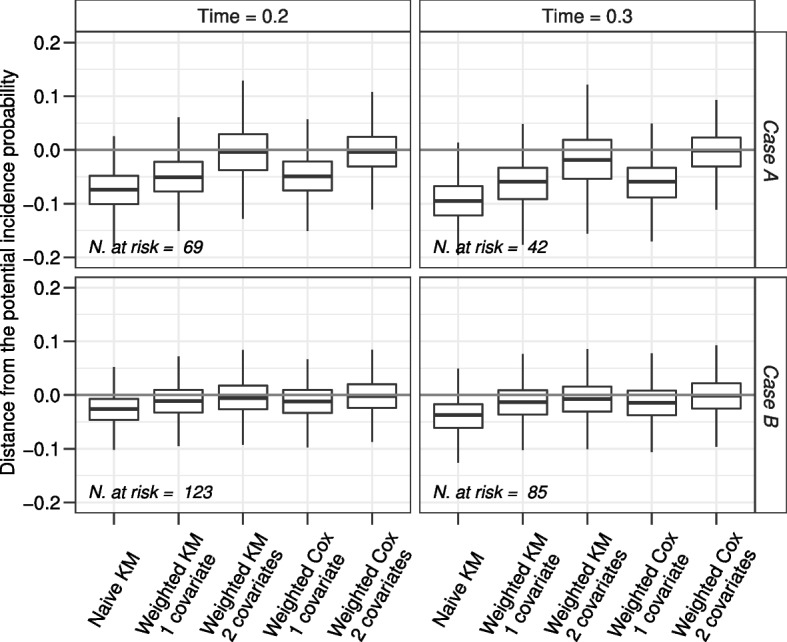
Simulation results for the variation of scenario 4 when $P(X_1=1)=P(X_2=1)=0.5$ (Case A) or $P(X_1=1)=0.3$ and $P(X_2=1)=0.1$ (Case B). The grey horizontal line is the reference null bias

Figure 8 shows results when the Bernoulli parameters change, named case A when $P(X_1 = 1) = P(X_2 = 1) = 0.5$ and case B when $P(X_1 = 1) = 0.3$ and $P(X_2 = 1) = 0.1$. As expected, in both cases only estimates obtained from the weighted KM and the weighted Cox model methods with the inclusion of both covariates are unbiased. Comparing the results of case A with the corresponding results of scenario 4 in Fig. 6 one may observe the greater variability due to the lower number of patients at risk of developing an event (AE or relapse). Comparing the results of case B with the corresponding results of scenario 4 in Fig. 6, the estimates of all methods are less biased.

**Fig. 9:**
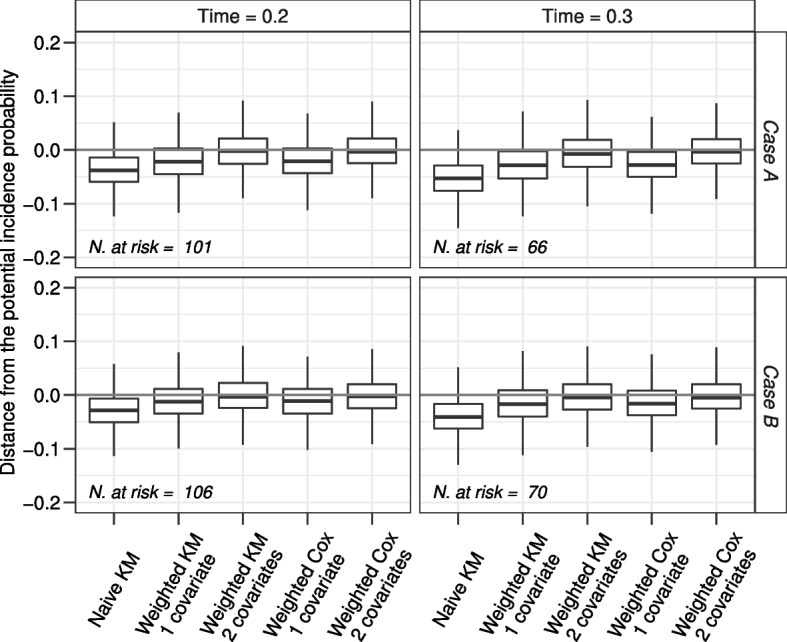
Simulation results for the variation of scenario 4 when fixed $X_1 = 0$ (or $X_1 = 1$), if $X_2$ changes, the hazard of relapse increases of 2 times (Case A) or of 1.5 times (Case B). The grey horizontal line is the reference null bias

Figure 9 shows results when the parameters of the exponential distributions from which the hazard of relapse was simulated change, named case A when $\lambda _{00RL}=2, \lambda _{01RL}=4, \lambda _{10RL}=5, \lambda _{11RL}=10$ and case B when $\lambda _{00RL}=2, \lambda _{01RL}=3, \lambda _{10RL}=5, \lambda _{11RL}=7.5$. Comparing the results with the corresponding results of scenario 4 in Fig. 6 one can observe that the lower is the hazard of relapse, the lower is the bias of the estimated incidence probability. Of note, in this simulation setting the bias obtained from the naïve KM censoring time to relapse reduces, but it is still the worst incidence estimator.

## Discussion and conclusion

In the majority of clinical studies on novel therapies with time-to-event endpoints, toxicity, related to the occurrence of AEs, is analysed with different estimators (such as crude proportions and rates) apparently disregarding the critical aspects that intervene due to the competing risks of AEs versus the primary endpoint event(s). In this work we propose solutions that relax the assumption of independence between the potential time to AE and the potential time to the efficacy endpoint event(s), thus allowing a proper estimate and interpretation of the cumulative incidence of AE. We addressed the particular case of non-fatal time-to-event efficacy endpoint. Of note, if failure was a fatal endpoint, the analysis of the occurrence of AE over time in principle could be carried out in the same way if the fatal endpoint is considered as a competing risk. However, the interpretation would be rather artificial. This is an obvious limitation of the method we proposed.

In the first part of this work we reviewed two different approaches starting from the type of clinical question when analysing AEs data and considering, for simplicity, one event for the efficacy endpoint, i.e. relapse. When the aim is the description of the observed occurrence of AE as first event in a competing risk framework (approach 1), treatment ability to protect from relapse has an impact on the chance of observing the AE due to the competing risks action. While the frequently presented crude proportion is not a function of time and does not properly account for censoring [5, 14], the Aalen-Johansen estimator of the crude incidence of AEs, commonly used for competing risks analysis [15], gives a proper estimate of the probability of treatment failure due to AEs over the course of time, where relapse acts as competing event (since AEs are counted only if observed as first events). The description of the observed occurrence of AE through the crude incidence together with the crude incidence of the efficacy endpoint enables to quantify the risks and benefits from the patient perspective [16]. Of note, the methods described in the previous citation can be useful to perform hypothesis testing on the two types of competing risks.

When the aim is the description of the potential occurrence of AE in relapse free patients, in a potential framework (approach 2), the epidemiological AE rate, which is not a function of time [4, 5], is often replaced by the Aalen-Nelson or Kaplan-Meier estimators of the cause-specific hazard of AE (as first event) and of the cumulative incidence of AE, regardless of relapse occurrence. However, the occurrence of relapse may exclude, not at random, patients from the risk sets on which the instantaneous rate of AE is calculated. This indirect patients selection operated by relapse, which is due to the dependence between the two potential times to AE and to relapse, leads to biased estimates.

One possibility to handle this dependence could be that of resorting to copula models treating the problem as a bivariate problem. This approach would go beyond our aim which is focused on a single marginal (that of the adverse event) and would require the specification of the type of copula. We think that resorting directly on a single marginal distribution is a more direct approach and has the advantage of not requiring a parametric specification of the type of copula [17].

We proposed alternative methods, such as weighted average survival probability (estimated either by the Kaplan-Meier estimator or by the use of the Cox model) and inverse probability of censoring weighting, and we proved through simulations that they overcome the problem due to the dependence between the potential times to AE and to relapse. In particular, we proved through simulations that one can handle patients selection in the risk sets, and thus obtain conditional independence between the two potential times, adjusting for all the observed covariates that induce dependence. Of note, we also show that, adjusting only for one observed covariate, thus ignoring the full dependence structure, gives anyway a less biased estimate compared to the naïve Kaplan-Meier estimator. The naïve Kaplan-Meier estimator, censoring time to relapse, is always biased, unless the hazard of relapse is independent from the covariates values. In a hypothetical scenario where all the covariates are observed, the weighted average incidence estimate obtained either non parametrically or by the Cox model and the inverse probability of censoring weighting would give an unbiased estimate of the incidence probability of AE or of the AE free survival curve. The same applies (data from simulations not shown) for the Aalen-Nelson estimator of the cumulative hazard of AE.

In addition, we pointed out that with the inverse probability of censoring weighting method one could obtain biased estimates when all the possible interactions between the observed covariates are not included in the model to estimate the weights (scenario 3 in Table 2). However, the inclusion of the interaction is not needed when the weighted Cox model is used, since conditional on the observed covariates, this model is robust in estimating the average incidence. Nevertheless, a limitation in the use of the weighted average survival method is given by the fact that it may be applied only in the presence of binary (or categorical covariates), since if the covariate is continuous it is impossible to identify the subgroups in which the incidence function can be estimated.

In general, our extended simulation protocol confirms that patients selection in risk sets is stronger and thus the bias is larger in the naïve KM estimator the higher is the imbalance in covariate values or the hazard of the competing event, relapse for given covariates [18].

Of note, we did not include the presence of right censoring in the simulation protocol since the estimators we investigated already can account for this additional complexity in the data.

Although clinical trials usually have an efficacy endpoint as a primary endpoint, safety analysis is always an important secondary endpoint, especially for non-inferiority trials. The approaches we proposed can be used for the comparison of the risk of developing adverse events depending on the treatment assigned and will be matter of future work.

## Data Availability

Available from the authors under request.

## Abbreviations

AE: Adverse event
AJ: Aalen-Johansen
AIEOP: Italian Association of Pediatrical Hematology and Oncology
ALL: Acute lymphoblastic leukemia
AN: Aalen-Nelson
pH: Potential hazard
pI: Potential cumulative incidence
pS: Potential survival function
CI: Crude cumulative incidence
CP: Crude proportion
CSH: Cause-specific hazard
IPCW: Inverse probability of censoring weighting
KM: Kaplan-Meier
ML: Maximum likelihood
ON: Osteonecrosis
PH: Proportional hazards
RL: Relapse
SE: Standard error
